# A Propaganda Index for Reviewing Problem Framing in Articles and Manuscripts: An Exploratory Study

**DOI:** 10.1371/journal.pone.0019516

**Published:** 2011-05-25

**Authors:** Eileen Gambrill, Amanda Reiman

**Affiliations:** 1 School of Social Welfare, University of California, Berkeley, California, United States of America; 2 Berkeley Patients Group, Berkeley, California, United States of America; University of Oxford, United Kingdom

## Abstract

**Objective:**

To determine the effectiveness of an index in increasing recognition of misleading problem framing in articles and manuscripts.

**Design:**

A propaganda index consisting of 32 items was developed drawing on related literature. Seventeen subjects who review manuscripts for possible publication were requested to read five recent published reports of randomized controlled trials concerning social anxiety and to identify indicators of propaganda (defined as encouraging beliefs and actions with the least thought possible). They then re-read the same five articles using a propaganda index to note instances of propaganda.

**Data source:**

Convenience sample of individuals who review manuscripts for possible publication and sample of recent published reports of randomized controlled trials regarding social anxiety in five different journals by different authors, blinded by author and journal.

**Results:**

Data showed that there was a high rate of propagandistic problem framing in reports of RCTs regarding social anxiety such as hiding well argued alternative views and vagueness. This occurred in 117 out of 160 opportunities over five research reports. A convenience sample of 17 academics spotted only 4.5 percent of propaganda indicators. This increased to 64 percent with use of the 32 item propaganda index. Use of a propaganda index increased recognition of related indicators. However many instances remained undetected.

**Conclusion:**

This propaganda index warrants further exploration as a complement to reporting guidelines such as CONSORT and PRISMA.

## Introduction

The propaganda index described in this article is designed to be used as a complement to reporting guidelines for reviewing manuscripts and articles. The flawed nature of peer review has long been of concern as illustrated for example by presentations at the International Congresses on Peer Review and Biomedical Publication.[1] The flawed nature of texts and other professional publications was one reason for the development of the process and philosophy of evidence-based practice.[2] A number of guidelines have been developed to enhance the quality of reporting such as CONSORT.[3] While such filters attend to methodological considerations, they do not address concerning problem framing such as the medicalization of common concerns.[4], [5], [6], [7], [8] This is especially unfortunate for readers who are not expert in an area who seek information related to life-affecting practice and policy decisions. Such censorship is a key form of propaganda.[9], [10], [11] The medicalization of problems includes various forms of disease mongering including transforming common problems-in-living into illnesses, viewing mild concerns as serious, exaggerating prevalence, use of words such as “insidious,” and claiming undertreatment and underdiagnosis.[7], [12], [13] This has become so extensive that a vigorous backlash has occurred.[4], [7], [12] The first international conference on the topic was held in Amsterdam in October 2010. Although experts in an area may recognize the absence of description of well-argued competing perspectives, for example the view that anxiety in social situations is a learned reaction,[14], [15] those who are not expert are unlikely to do so.

## Methods

### Development of the index

**Figure 1:**
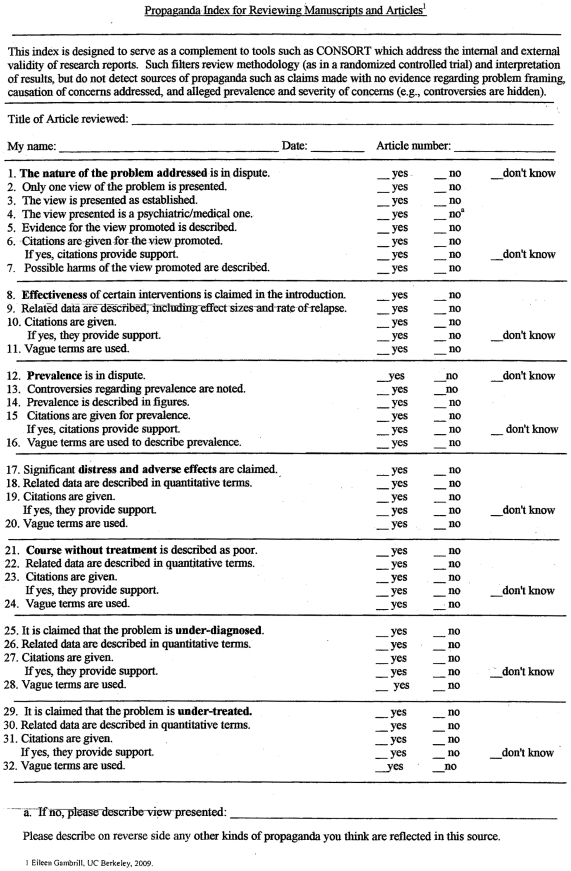
Propaganda Index.

An index consisting of 32 items divided into seven categories was developed drawing on related literature on propaganda, peer review and problem framing (see Figure 1). This literature pointed to the following content regarding problem framing and evidentiary issues. The first category pertained to the nature of the problem addressed: Is it in dispute? Is only one view presented? Is this view presented as established? Is a psychiatric/medical view presented? Is evidence for the view promoted described? Are citations given? If so, do they provide support? Lastly, are possible harms of the view promoted described? Other sections included claims regarding effectiveness of interventions; claims regarding prevalence; claims regarding significant distress and adverse effects of the problem addressed; claims regarding course without treatment; claims of under-diagnosis; and claims of under-treatment. The latter three are indicators of disease mongering.[7], [12], [13] (See Appendix A for the instrument.) Respondents were also requested to indicate whether evidence was provided for claims (e.g., data described in quantitative terms, effect sizes), whether vague terms were used and whether citations were given and, if so, whether these provided support (yes, no, don't know).

### Data Source

Five recent reports of randomized controlled trials concerning social anxiety disorder were selected representing five different journals and different authors.[16], [17], [18], [19], [20] A convenience sample of 17 subjects who review manuscripts for publication was selected. All had a doctoral degree but none specialized in the area of social anxiety.

### Procedure

Upon agreement to participate, each respondent received an envelope containing a brief description of propaganda defined as encouraging beliefs and actions with the least thought possible [**9**] and was asked to read the five articles included in the package (blinded by author and journal in which they appeared). They were asked to focus on the introduction rather than the methodology and to circle directly on the article, any indicators of propaganda they saw and to describe why they thought each was a sign of propaganda. The instructions informed them that "This index is designed to serve as a complement to tools such as CONSORT which address the internal and external validity of research reports and interpretation of results."

They were asked to write "none" at the top of the page if they thought there were no indicators in an article. When finished, they were requested to place the five articles in the stamped addressed envelope enclosed and to remove a second set of the same articles as well as to open a smaller envelope containing ten copies of the index and to use the first 5 copies to again review the 5 articles, this time using the propaganda index. They noted the article number on each respective form and then mailed the first set of five articles plus the copies of the five index forms to the first author. They were requested to keep the second set of five articles as well as the second set of propaganda indices and to again review the articles using their second set two weeks later and to mail these back to the first author. This served as a reliability check.

### Data Analysis

**Figure 2:**
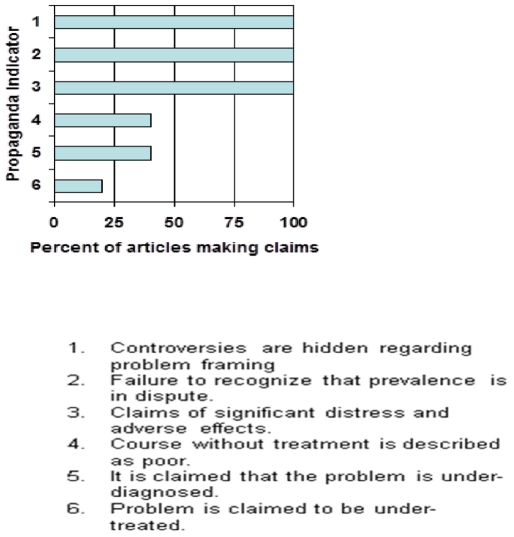
Censorship and claims making regarding problem framing in 5 published RCT's on social anxiety (as identified by the author and Amanda Reiman, PhD).

The first author reviewed each article to identify indicators of propaganda. A high rate was found: 117 out of 160 opportunities over all five articles. Indicators included vagueness, lack of documentation and disease mongering (see Figure 2). This review served as a criterion.

Examples of rhetoric regarding problem framing can be seen below.

- “Social phobia is a common and disabling anxiety disorder associated with considerable social and occupational handicap that is unlikely to remit without treatment.”
- “Generalized social anxiety disorder is a chronic and insidious psychiatric disorder that first received widespread attention during the 1980′s. Social anxiety disorder has an early onset, typically between 14 and 16 years of age, and subsequently follows a chronic course that persists well into adulthood. Spontaneous recovery is possible, but it occurs gradually and only in about half of all sufferers.”
- “Social phobia (also known as social anxiety disorder) is associated with substantial impairment in quality of life (Safren, Heimberg, Brown & Holle, 1997) and is highly prevalent (Furmark, 2002). As evidenced by several trials, there are effective psycho-social treatments for social phobia (Heimberg, 2001). However, far from all sufferers seek treatment (Baldwin & Buis, 2004).”

## Results

**Figure 3:**
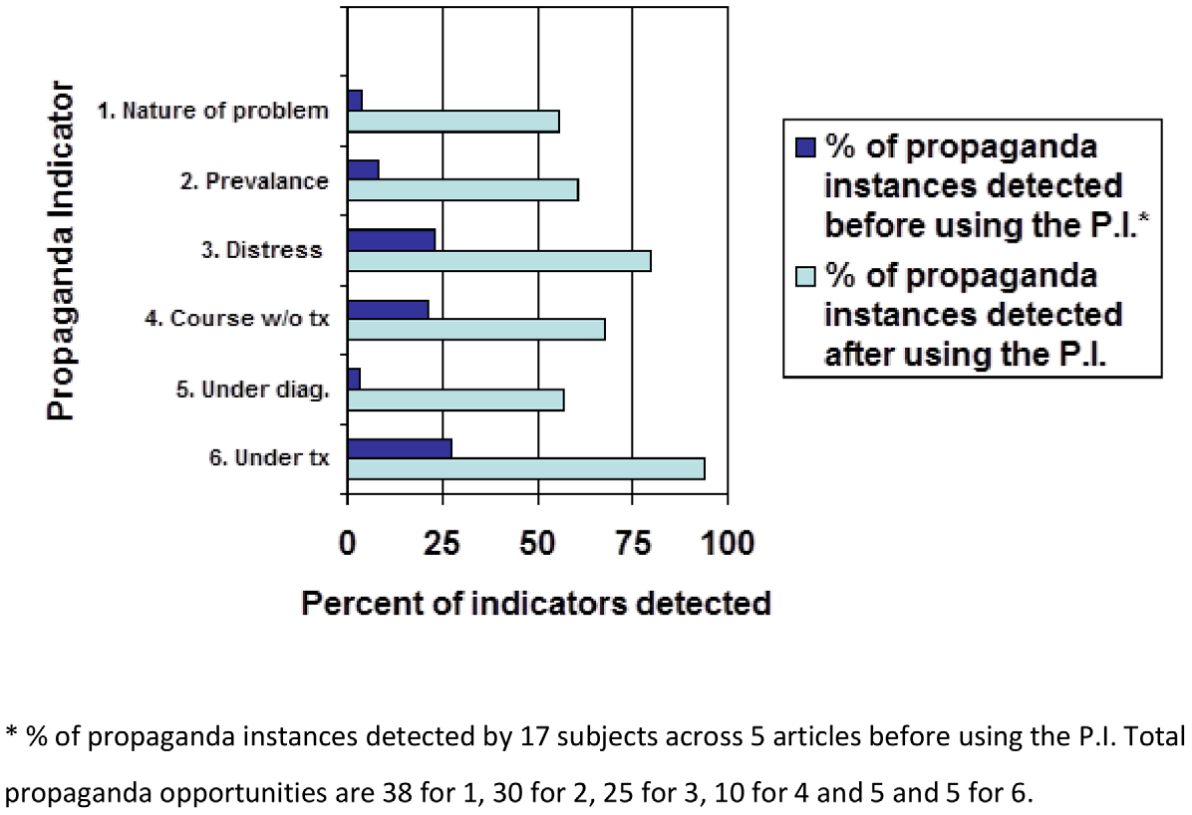
Propaganda detection before/after using the P.I.

The Master P.I. was used to determine the number of opportunities to spot propaganda across the five articles. All five RCT's reflected hiding of controversies regarding problem framing, failure to recognize that prevalence is in dispute and claims of significant distress and adverse effects (see Figure 2). The second author independently reviewed the five articles. Inter-rater reliability between the first and second author was .88. Then, the data from the articles submitted by each participant before using the index and after using the index were analyzed to determine the percentage of propaganda detected by participants before and after using the P.I. Results indicate that participants were able to detect propaganda at a higher rate after using the P.I. (see Figure 3). For example, out of a possible 38 propaganda indicators concerning the nature of the problem presented across five RCT's, participants detected an average of 1.5 indicators before using the Propaganda Index, and an average of 21.3 indicators after using the index. Similarly, participants identified an average of 2.4 out of 30 indicators concerning reported prevalence before using the Propaganda Index, and an average of 20 indicators after using the index. Furthermore, before and after using the propaganda index, the dimension of under-diagnosis was most commonly missed by participants. The dimension of under-treated saw the most improvement in detection after using the index, raising the rate of detection by 67% (average detection of 1.3 items out of 5 before the index, and 4.7 items out of 5 after the index). The mean percentage of indicators detected over all five articles before use of the index for the 17 subjects was 4.5 percent. This increased to 64.3 percent following use of the index. Test-retest reliability for subjects was .89 (range .82–.97).

## Discussion

Major advances have been made in creating guidelines designed to enhance reporting of research. Examples include STARD, MOOSE, CONSORT, TREND and PRISMA. There has been increased transparency regarding conflicts of interest created by funding of authors by pharmaceutical and biotech companies and other kinds of financial ties with such industries such as owning stock.[21] However, there is often (if not typically) silence in research reports in journals regarding controversies about problem framing. This silence (this partiality in the use of evidence by hiding well-argued alternative views and related evidence) is a hallmark of propaganda. Propaganda is defined as encouraging beliefs and actions with the least thought possible. [9] This silence serves to maintain and advance questionable practices such as translating common problems-in-living into mental illness and hiding related controversies. It deprives readers of an opportunity to be informed. This is especially true in psychiatry and allied professions such as clinical social work and psychology in which the medicalization of problems has been so successful. This success has not gone uncritiqued as illustrated by the resultant backlash. What is already known on the topic: 1) Translating common problems-in-living into mental illness and other forms of disease mongering is common; 2) Little or no attention is paid to problem framing in reporting guidelines such as CONSORT. What this study adds: 1) Draws attention to propagandistic framing of problems in reports of RCTs regarding social anxiety; 2) Suggests the need to include questions encouraging critical review of problem framing in filters such as CONSORT guidelines; 3) Suggests that even when prompted, reviewers miss many indicators of propagandistic framing of problems.

Our concern here is the large body of work in which a “mental illness” framing is presented as true and uncontroversial in reports of research, for example RCTs regarding “social anxiety.” That is, there is no mention of well-argued competing perspectives and related evidence, for example, the view that anxiety in social situations is a learned behavior which can be decreased by arranging new learning opportunities (without medication).[14], [15] Red flags for hiding competing well-argued views include phrases such as “Every one knows …” “It is clear that …” “It is obvious that …” “It is generally agreed that …” This kind of unchallenged repetition encourages the woozle effect; if we hear something enough times we assume that it is true. A mental illness perspective is also promoted in direct-to-consumer advertising and in the wider media rendering silence regarding well-argued competing views even more pervasive.[13], [22] This exploratory study highlights the prevalence of propagandistic problem framing including disease mongering in published descriptions of RCTs concerning social anxiety and the utility of a propaganda index in increasing readers' detection of related indicators. However, many subjects still missed many important indicators.

The propaganda index is designed to serve as a compliment to methodological filters in reviewing the quality of manuscripts and articles. We suggest that reviewers and editors be required to consider more carefully, from an evidentiary and conceptual point of view, the framing of concerns addressed in reports of research. Recommendations for reviewers and editors include requiring authors to reveal rather than hide controversies, for example to accurately describe well-argued alternatives to views promoted. This would take one sentence such as: “An alternate view is that anxiety in social situations is a learned reaction created by an unusual learning history,” then cite relevant references. We assume that journal editors sent manuscripts of their articles to “experts” in the area of social anxiety. Clearly neither reviewers or editors requested authors to note controversies regarding problem framing. Authors should be required to avoid weasel words such as “common” (actually give figures) and disease mongering terms such as “insidious.” They should be required to describe quantitative data related to claims made (e. g., effect sizes, and size of correlations in place of vague terms such as “most,” “few”). Next steps include checking citations used: do they provide evidence for claims made? Preliminary inspection indicates that textbooks are sometimes referred to to support empirical claims. Secondly, correction of problems in the Propaganda Index is necessary, for example some items are not applicable after a “no” answer. Thirdly, we plan to explore the correlation of propaganda regarding problem framing with quality of RCT using critical appraisal tools such as the JAMA User guides. Further exploration is needed with increased sample size. Also, what results would be found if we sent these same five articles to experts in social anxiety? Would the results be similar? Lastly, an item analysis should be carried out to determine whether the index can be shortened without loss of value.
